# Addressing Vaccine Hesitancy in China: A Scoping Review of Chinese Scholarship

**DOI:** 10.3390/vaccines8010002

**Published:** 2019-12-20

**Authors:** Ronghui Yang, Bart Penders, Klasien Horstman

**Affiliations:** Department of Health, Ethics and Society, Care and Public Health Research Institute (CAPHRI), Maastricht University, PO Box 616, 6200 MD Maastricht, The Netherlands; b.penders@maastrichtuniversity.nl (B.P.); k.horstman@maastrichtuniversity.nl (K.H.)

**Keywords:** vaccination hesitancy, China, vaccine safety, governance

## Abstract

Despite the well-developed Chinese National Immunization Program, vaccine hesitancy in China is rising. As part of the response, Chinese scholars have studied determinants and proposed solutions to vaccination hesitancy. We performed a scoping review of Chinese literature (2007–2019), drawn from four Chinese databases. We mapped relevant information and presented a systemic account of the proposed determinants and responses to vaccine hesitancy in China. We identified 77 relevant studies that reveal four approaches to vaccine hesitancy. Most Chinese studies define vaccine hesitancy as a problem of vaccine safety and vaccine incident response and place accountability on the level of governance, such as regulation deficits and inappropriate crisis management. A first minority of studies tied vaccination hesitancy to unprofessional medical conduct and called for additional resources and enhanced physician qualifications. A second minority of studies positioned vaccination hesitancy as a problem of parental belief and pointed to the role of media, proposing enhanced communication and education. Chinese literature ties vaccine hesitancy primarily to vaccine safety and medical conduct. Compared to international research, parental concerns are underrepresented. The Chinese context of vaccination scandals notably frames the discussion of vaccination hesitancy and potential solutions, which stresses the importance of considering vaccination hesitancy in specific social and political contexts.

## 1. Introduction

Four decades after the start of China’s National Immunization Program (NIP) for children in 1978, a complete vaccine chain and a strict regulation system up to the WHO standards have been established in the country [1,2]. The Chinese vaccination program distinguishes between Category 1 and Category 2 vaccines. Category 1 vaccines are provided for free for all children until 14 years, and the use of these vaccines is—although not mandatory—considered a social duty [3]. Category 1 vaccines include vaccines to prevent diseases, such as the hepatitis B vaccine, Bacillus Calmette–Guérin vaccine, polio vaccine, diphtheria–tetanus–pertussis (DTP) vaccine, and MMR vaccine. Children are vaccinated at the local centre for disease control (CDC) or affiliated agencies before they enter school, join the army, or go abroad. Category 2 vaccines are optional vaccines and that have to be paid for by the parents. The vaccines in this category include, for instance, vaccines to prevent human papillomavirus (HPV), mumps, rubella, pneumococcus, and rotavirus. The coverage of Category 1 vaccines is higher than that of Category 2 vaccines and, sometimes, the authorities consider moving a vaccine from Category 2 to Category 1—as is currently the case with the pneumococcal vaccine—to reduce suffering among children [4]. According to a report by the national CDC, the Category 1 vaccination rate in China in 2019 was higher than 90%, which is among the highest in the world [3]. 

A systematic review of global studies on vaccination hesitancy [5] identified only one Mandarin study published in 2007 and two studies of vaccination hesitancy in China published in English journals. In [6], where the authors aimed to map vaccine hesitancy globally on the basis of interviews with immunization managers, no Chinese interviewees were included. Nevertheless, vaccine hesitancy is considered an important problem in China [4,7]. In the last decade, a series of reports about the serious side-effects of vaccination has increased vaccination hesitancy and distrust in the immunization program [8]. In 2010, media outlets reported on vaccination-induced disability and questioned the death of nearly a hundred children due to the uptake of invalid vaccines that were exposed to high temperatures [9]. In 2014, it was reported that eight babies died after hepatitis vaccination in southern China [9], and in 2016, a large number of expired vaccines circulated in Shansong Province caused public anxiety [10]. In 2018, the Changsheng company falsified records of vaccine production, and as a result, children were injected with unqualified vaccines, leading to panic among parents nationwide [11]. These incidents have eroded people’s confidence in China’s NIP. According to [12], after the Hepatitis B vaccine crisis of 2014, 30% of parents started doubting vaccination. In a survey [13] conducted in a city in the Shandong province, 77.78% and 87.78% of parents expressed their doubts about Category 1 and 2 vaccinations, respectively, after the Shandong vaccine crisis of 2016. According to [14], after the Changsheng vaccine crisis of 2018, 93.4% of parents in Yangzhou had less trust in Chinese vaccines. In line with this reduced trust, the vaccination rate dropped. Affected by the 2014 Kangtai hepatitis vaccination problems, the hepatitis vaccination rate dropped by 30% in 10 provinces. In 2016, the vaccination rates for a few Category 2 vaccines, especially the rotavirus vaccine, decreased massively. The rates of rotavirus vaccine, *Hemophilus influenzae* type b vaccination and varicella live attenuated vaccination decreased by 20.49%, 40.60%, and 28.26%, respectively, compared to those in 2015 [15]. In addition, owing to the effects of these vaccine safety crises, the Category 2 influenza vaccination rate stood at only 2% in 2018 [16].

Vaccine hesitancy was defined by the WHO in 2015 as a delay in acceptance or refusal of vaccination despite availability of vaccination services [6]. Building on the definition of vaccine hesitancy, the WHO Strategic Advisory Group of Experts (SAGE) drafted a “Model of determinants of vaccine hesitancy” in 2012, organized around three key domains. These are 1) contextual influences—including historic, social-cultural, environmental, health system/ institutional, economic, or political factors; 2) individual and group influences—including influences arising from personal perception of the vaccine or influences of the social/peer environment; and 3) vaccine and vaccination-specific issues, which are directly related to the characteristics of the vaccine or the vaccination process [5,6]. Most studies have focused more on individual and group influences and contextual influences than on vaccine-related issues [17,18,19]. Accordingly, education of, communication with, and information dissemination to parents are collectively considered an important strategy globally. Studies on vaccination hesitancy in China published in international journals paint a similar picture. For instance, it has been argued that the public lacks adequate vaccine knowledge and is unable to recognize the risks of vaccination [20]. The authors of these study advised that the Chinese state should launch educational campaigns to improve parental awareness and knowledge of vaccination. Along similar lines, researchers [8,9,21] have argued that media coverage of vaccine incidents have amplified public concerns and fuelled vaccine hesitancy. According to [9], to counteract these media influences, an online communication mechanism should be established by the state to engage with the public in a timely manner, avoid misinformation, immediately launch an investigation to determine the clinical situations of causality, and monitor public confidence. 

However, other internationally published studies on vaccination hesitancy in China have not focused on parental considerations and the influences of media on parental decisions but instead on the governance of vaccine safety. In [11,22,23], researchers have argued that pharmaceutical enterprises are responsible for the vaccine-related scandals in China and that the government should reform the supervisory model to strengthen regulation of the vaccine chain from production to market and to alleviate public anxiety. Similarly, in [24], the authors argued that the surveillance system of Adverse Events Following Immunization played a major role in stimulating distrust because the system was unable to collect sufficient data about the side effects of vaccination and underreported the social impact of such side effects. According to [24], the government should strengthen its surveillance capacity and develop a new, active surveillance system.

To provide more insights into vaccination hesitancy in China, we aim to analyse how vaccination hesitancy and the governance of vaccination hesitancy in China are studied in Chinese academia. Most studies on the governance of trust in vaccination in Chinese academia focus either on a specific case study [25] or on a subset of governance strategies (e.g., crisis management, accountability of enterprise) [26]. A systematic exploration of studies on this topic is yet to be conducted. To address this gap, we conducted a scoping review of Chinese publications. We analysed a specific set of publications by focusing on three main questions: How is the problem of vaccine hesitancy defined? How are the responsibilities for this problem allocated? What are the proposed solutions?

## 2. Materials and Methods

To perform a scoping review, we followed the framework proposed by Arksey and O’Malley [27], supplemented with the PRISM-ScR extension for scoping reviews [28] in as much as possible given the different information contained in Chinese databases and research papers. This framework can be divided into five stages: (1) Identification of the research questions; (2) identification of relevant studies; (3) study selection; (4) data charting; and (5) reporting the findings.

### 2.1. Identifying Research Questions

This scoping review aims to study how Chinese scholars have analysed the governance of the Chinese NIP: How have they defined the problem, how have they assigned responsibility, and which solutions have they proposed? The purpose is to map and understand the Chinese academic and professional debate about vaccine hesitancy and to highlight areas for further analysis.

### 2.2. Identifying Relevant Studies

To identify relevant studies, we focused on scholarly and grey literature about the governance of vaccine hesitancy in China published between 2007 and September 2019. The starting point was set to 2007 because we did not find any research on vaccine hesitancy published before 2007. We consulted four China databases, namely China National Knowledge Infrastructure (CNKI), Baidu Scholar (BS), Wanfang (WD), and Chongqing (CVIP), which index academic and professional articles, government reports, and public commentaries. Chinese databases employ slightly different demarcation criteria for scholarship and index some semi-journalistic publications. Since Chinese research infrastructure considers them to qualify as scholarship, we did not exclude them. These four databases are widely used by Chinese scholars, and they are considered authoritative by academics and professionals. We searched these databases on scope, including the title and abstract. Aiming to obtain insights into the scholarly debate on the governance of vaccines and concerns surrounding vaccination in China, the following search terms were selected: vaccine concern, vaccination concern, vaccine incident response, safety issue and cause, enterprise production, media report, supervision system, medical staff, risk assessment, public participation, assessment criteria, risk communication compensation system, and countermeasures.

### 2.3. Selection of Relevant Studies

Following an electronic search, titles and abstracts were screened and full articles were reviewed to determine whether they met the eligibility criteria. The inclusion and exclusion criteria are listed in Table 1.

### 2.4. Charting Data

We recorded all relevant publications in a spreadsheet, including information about authors, publication year, authors’ expertise, disciplinary focus, category of vaccine hesitancy (demarcating four sets of problem/responsibility/solution conceptualisations) (see Table A1). We screened the articles based on how the authors defined the problem of vaccine and vaccination concerns, how they assigned responsibility for the problem, and which measures they proposed. 

### 2.5. Presentation of Results

We present the results by organizing them into five sections: How many relevant articles were selected? How do scholars define the problem of vaccine hesitancy? How did they assign responsibility for the problem? Which measures did they propose? How did they respond to new policies? 

## 3. Results

### 3.1. Selected Articles

**Figure 1:**
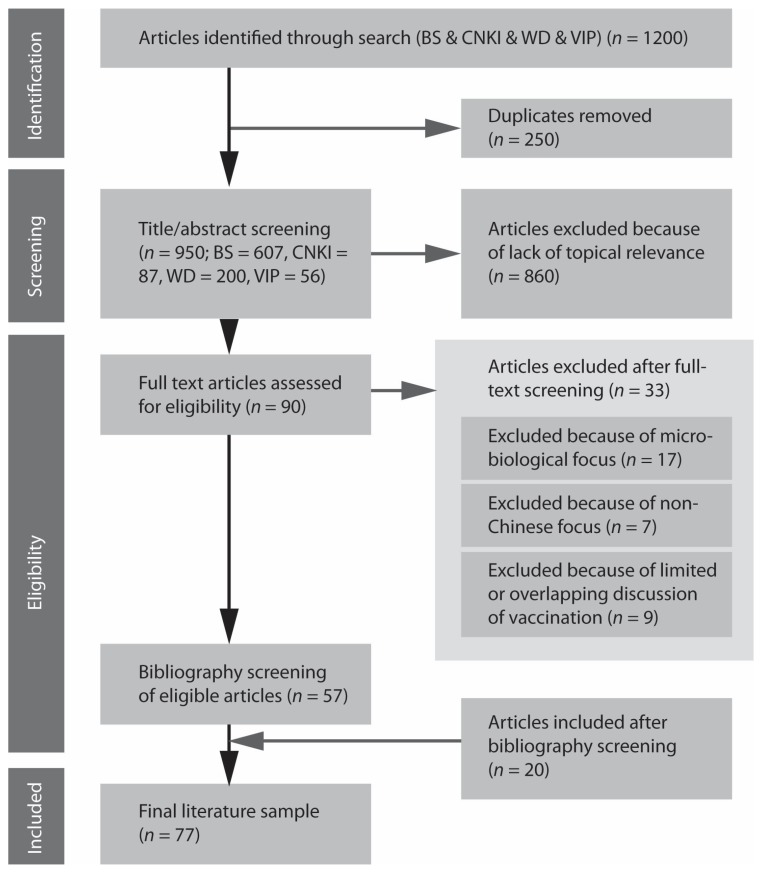
PRISMA chart.

We identified 1200 papers, out of which 250 papers were excluded because they were repeatedly presented in four databases, resulting in 950 papers (see Figure 1). After title and abstract screening, 90 papers were included for full text screening. Out of the 90 articles, 32 papers were excluded for the following reasons: 17 articles focused on the analysis of microbial vaccines, 7 articles studied foreign vaccines, 5 only mentioned vaccines in the conclusions, and 4 had overlapping content. In case of overlap or duplication, we included the oldest publication in the set. In addition, 20 new articles which were not part of the original search results (*n* = 1200), were included after bibliography screening, as a result of divergent terminological use. Finally, 77 relevant articles were identified for inclusion in the review. 

### 3.2. Defining the Problem of Vaccine Hesitancy

**Figure 2:**
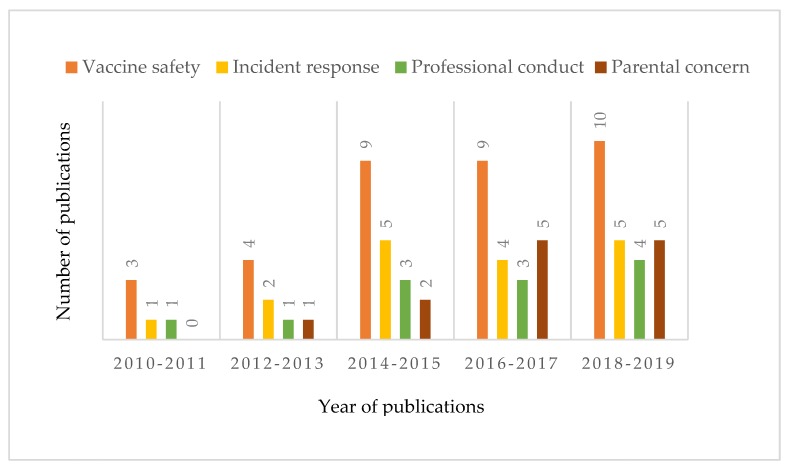
Distribution of publications on the governance of vaccine hesitancy across four categories during 2010–2019. Categories were assigned based upon problem articulation, solution proposals, and the allocation of responsibility (see Section 3.2).

Among the selected articles, we distinguished four different approaches to vaccine and vaccination hesitancy, namely vaccine safety (*n* = 35), vaccine incident response (*n* = 17), professional conduct (*n* = 12), and parental concerns (*n* = 13) (see Figure 2). This categorisation is based on the three research questions, focussing on the description of the problem, the group assigned responsibility for that problem, and the proposed solutions. Most of the studies focused on unsafe-vaccine-induced hesitancy, and an increasing number of studies on this topic were published between 2010 and 2019. Although the number of studies on parental concerns has increased gradually over the last seven years, such studies were a minority. Most articles on incident response were published between 2014 and 2019, which may be affected by the hepatitis B vaccine incident of 2014, illegal vaccine event of 2016 in Shandong, and Changsheng vaccine scandal of 2018.

#### 3.2.1. Vaccine Safety

Most scholars have argued that vaccine hesitancy can be ascribed to unsafe vaccines. Enterprises adopted illegal production techniques and falsified production records, leading to huge risks related to vaccine safety [29,30,31,32]. This resulted in parents believing that vaccines are unsafe and may harm the health of their children [14]. According to epidemiologists, unqualified refrigeration decreases the efficacy of vaccines, which is unfavourable for disease prevention and reduces public trust in vaccine safety [33,34,35,36,37,38,39,40]. 

Scholars have pointed to illegal corporate production, supervision model deficits, state–business collusion, and bureaucratic production system as routes for understanding the presence of unsafe vaccines. Some social scientific studies have indicated that driven by commercial profits, enterprises produced problematic vaccines, which were used to inoculate many children nationwide [41]. When this fact was disclosed by the media, companies responded by suppressing evidence and shirking responsibilities, and according to the authors, they should be punished severely for these acts [26,41,42,43]. Moreover, a couple of public policy scholars have stressed that the bureaucratic production system leads to price squeezing in vaccines, and to obtain profits, enterprises must produce vaccines illegally [30,44].

Furthermore, sociologists and public policy experts [45,46] have argued that local state leaders who collude with companies to further their economic interests and for self-career advancement are responsible for problematic vaccines. They administer the distribution of state resources and prioritize the disbursal of loans to enterprises via power and rent-seeking, which provokes commercial bribery [46]. Moreover, to spur local economic development and favour the promotion of their political careers, local leaders connive in corporate violations, which weakens the governmental supervision capacity to some extent [45].

A handful of scholars in the fields of public policy have proposed that supervision mode deficits are to blame for problematic vaccines. They have argued that the Chinese horizontally segmented regulatory model with less collaboration among supervisory bodies leads to power overlaps and vacuums, which hinders strict supervision of the vaccine industrial chain from production to market [30,31,44,47,48,49]. Meanwhile, owing to reformation of the vertical hierarchical regulatory system in 2012, daily supervision tasks are top-down reallocated at the grassroots supervisory departments. Consequently, grassroots governments that lack experienced medical staff and advanced technologies have been unable to complete the highly technical supervision tasks, thus weakening the state’s capacity to enforce regulations pertaining to vaccine safety [50]. 

#### 3.2.2. Vaccine Incident Response 

Along similar lines, several social policy researchers have noted that public distrust should be attributed to failed crisis management efforts on the part of the state and experts, for example, risk assessment and risk communication of vaccine incidents. The lack of public participation and lack of information transparency in risk assessment [42], as well as suspicions about state–expert conspiracy, have reduced the credibility of expert risk assessment [51,52,53,54,55]. In [52], the authors have argued that a qualification entry setting restrains the participation of the lay public and that experts privatize data and keep the process non-transparent in an attempt to reduce disputes and pressure from the society. Moreover, they argue experts conventionally give voice to the public interest and public values in China. Yet they are now subject to politics, which has spurred distrust [52,53]. 

The lack of systematic risk communication is related to public distrust as well. Journalists have argued that because local officials are concerned about accountability, they tend to restrain the dissemination of negative information when crises occur and have little regard for information transparency and risk communication in an attempt to reduce social concerns [54,55,56,57]. In addition, there are no special personnel and specific policies and strategies to support systematic communication between the state and public, leading to increased distrust of the state [58,59,60]. 

#### 3.2.3. Professional Conduct of Vaccination

In addition to vaccine safety and incident response, Chinese scholars have related the professional conduct of vaccination personnel to vaccination concerns. Epidemiologists have argued that pre-vaccination contraindication intimation and post-vaccination observation for 30 min could reduce the occurrence of vaccination side effects and increase trust in the vaccination process. However, in practice, doctors exhibit little regard for pre-vaccination screening and post-vaccination observation, stressing instead on completion of the vaccination task [61,62]. In addition, doctors are impatient and not very responsive to questions during vaccination, which causes the public to be dissatisfied with medical services [63,64]. Furthermore, the use of relatively old technologies at the grassroots level, such as old refrigeration systems and unqualified information traceability mechanisms, are to blame for reduced trust [1,32,65,66,67].

#### 3.2.4. Parental Concern 

Other scholars have pointed out the contribution of parents to increased vaccination hesitancy. A public health expert has argued that declined trust in vaccination can be ascribed to the cognitive biases of parents [68]. Parents consider vaccination as highly risky and keep doubting vaccines, which results in public distrust and decreases the rate of Category 2 vaccination [10]. According to epidemiologists [16], owing to parental hesitancy, the average annual flu vaccination rate in China stand at only 2%–3%. In addition, in 2017, 40.5% of parents in western China were hesitant to vaccinate their kids against EV71 [69]. However, several CDC experts have argued that imprecise reports in the media stimulated parental distrust: eye-catching titles, such as “toxic vaccine,” “life-killing vaccine,” and “fearing and screaming” further stimulated parental risk perception and parental concerns [12,70,71]. 

Epidemiologists have also [17,72,73,74,75] argued that parents do not have adequate cognitions on the hazards of epidemics and the benefits of vaccination, resulting in the distrust of vaccines. In [69,76], the authors have emphasized that the lengthy queues for vaccination, bad attitude of medical staff, and lack of communication increased public dissatisfaction in vaccination. In addition, in [68,77], the authors have focused on the poor accessibility of vaccines because of the high prices of Category 2 vaccines, or the need to travel far affected the registration system, since children in some places were required to accept Category 1 vaccination in their town of birth. Researchers have highlighted the prevalence of vaccination hesitancy among medical staff, which leads to negative publicity about vaccines [65].

### 3.3. Defining Solutions for Reducing Vaccination Hesitancy

In line with the different ways in which Chinese academics have defined the problem of vaccination hesitancy, studies have focused on different strategies to deal with the problem. A dozen studies have called for a centralized model to supervise vaccination safety. Before 2000, vaccines were distributed top-down by the state, and local governments were responsible for vaccine hygiene regulation. Owing to strict political control over the vaccination infrastructure at that time, adverse incidents related to vaccines rarely occurred [78]. In 2010, in response to the expansion of the vaccination program and the increasing complexity of the vaccine chain, a segmented model was introduced: Five different supervisory bodies assumed responsibility for vaccine safety in different phases of the vaccine chain [26,30]. However, with the emergence of vaccine incidents, the segmented model was again centralized to tackle the power vacuum and overlap [49,79,80]. Moreover, a strict administrative accountability mechanism was introduced to restrain illegal production and official malpractice [25,39,45,46,81].

In addition to improvements to the supervisory model, some scholars have suggested that the system for controlling the cold chain should be improved as well. Public health experts have advised that vaccine-related risks be classified, equipment updated, standard operation procedure regulation (SOP) enacted, regular risk assessment of refrigeration carried out, and information traceability through good data management established [15,67,82].

Several studies have emphasized that professional medical conduct should be regulated. A group of public health experts have proposed that the vaccination standards be raised, such as pre-vaccination screening and post-vaccination follow-up [59,83,84]. During vaccination, communication between doctors and parents could be improved, information could be shared, and professionals could be more responsive to questions and doubts [85]. To increase the accountability of doctors [83,85,86], information systems for recording data, such as the health status of a child and vaccination procedure, should be improved. Other scholars have focused on creating entry qualifications for doctors and implementing regular training and annual assessments for doctors to improve their skills [65,87,88]. Advanced refrigeration equipment, medicinal freezers, and alarm devices for temperature should be adopted at hospitals to ensure vaccine safety [67,84].

A handful of scholars have argued for a so-called participatory turn during incident response. Sociologists [51,54,55] have suggested that risk assessment procedures should allow stakeholder participation, and discussion should be conducted with these stakeholders to ensure the fairness of risk assessment and prevent data falsification by experts. Several scholars have proposed a systematic risk communication system to facilitate expert–public communication [59,89]. Furthermore, several scholars have highlighted the importance of adequate compensation mechanisms to tackle crisis-led public distrust. They have argued in favour of expanding the scope of compensation to cover all vaccination-reaction-led causalities, simplifying compensation procedures, and introducing commercial insurance to increase compensation [57,90,91,92,93,94].

Some scholars have addressed parental concerns and beliefs. They have suggested that the state should regulate the media to control online rumours [70,71]. Some have argued for adequate education of the public to mitigate personal risk perception [16,73,74,75,76], expanding the scope of free vaccines to reduce family expenses, and compulsory vaccination to tackle vaccination refusal [13].

### 3.4. Implementing New Policies

Some Chinese studies on vaccination hesitancy have put forth new policies to reduce the public’s distrust in vaccination. Several studies have analysed the centralization of the segmented model to overcome the deficits in the segmented model. The authors of [81] indicated that the centralized model makes the Food and Drug Administration responsible for vaccine production, storage, circulation, and marketing, and the Health Department responsible for vaccine safety in medical institutions. Following this, the authors of [39] stated that a strict top-down accountability system, public complaint mechanism, and specific punishment mechanisms were installed in 2015 to hold officials and manufacturers accountable. According to an analysis of [79], in 2018, a centralized agency called the Market Supervision Administration, which was formed by merging the Food and Drug Administration, Industrial and Commercial Administration, and Health Administration, was established nationwide to prevent shirking of the responsibility for implementing vaccine safety regulations and to optimize the allocation of supervisory resources.

A few studies have analysed the “Draft of Vaccine Regulation Law” that was published by the state in 2018. This draft has sparked scholarly discussions in China. The “Law of Vaccine Regulation in China” was approved in the 11th meeting of the Standing Committee of the 13th National People’s Congress on 29 June 2019, and will be implemented on 1 December 2019 [94]. Most scholars have assessed this new law rather positively. Both public policy experts and public health experts [30,65,95,96] have argued that this law underlines the importance of adequate communication with the public. Additionally, according to an analysis in [91], the law prescribed a compensation system for vaccination victims who exhibited an abnormal vaccination response. Furthermore, in [93], the authors argued that the state encourages commercial insurance providers to provide more compensation to victims and that it complements the public compensation system in an important way. The attempt made in the “Draft of Vaccine Regulation Law” to raise the quality standards of medical doctors was also received positively. The authors of [85] stated that the law will oblige doctors to inform parents about contraindications before vaccination and monitor post-vaccination effects, and in [55], it was argued that the medical institutions and doctors involved in vaccination should have specific qualifications. 

Some Chinese scholars have clarified that although new policies were introduced to deal with vaccination hesitancy, the implementation of these policies was hampered. A journalist [97] emphasized that it is difficult to hold medical doctors accountable in cases of malpractice because the criteria for malpractice are rather subjective. Although regulations state that doctors should bear certain responsibility for medical accidents, there are no criteria to define the severity of medical incidents or the degree of doctors’ responsibility. Complementarily, a public policy expert [98] stated that the system of accountability is very complex: Different bodies play different roles in the organization of accountability for medical incidents, and a lack of collaboration among these bodies causes tensions related to accountability. The local government, health bureau, court, social supervision committee, and medical ethics committee govern political accountability, administrative accountability, legal accountability, social accountability, and professional accountability, respectively. As a result, medical staff are confused as to whom they are accountable to and what they are accountable for [98]. 

Some sociologists [51,53,55] have argued that public distrust in vaccines has increased because of experts’ subjective judgment and decision-making without incorporating public values and that to reduce this distrust, different stakeholders should be engaged in risk-assessment procedures. According to a journalist [56], experts perform risk assessment within a rather narrow biological, chemical, and physical scope, and they are unable to incorporate novel viewpoints and considerations lying outside their framing of the problem. A CDC expert [12] argued that engaging lay people who are unable to conceptualize the hazards in risk assessment will hamper expert risk assessments. Others disagree with this line of reasoning. In [55], it has been argued that risk communication, instead of being a democratic dialogue, is dominated by experts who communicate top-down and regard the public as ignorant and irrational.

## 4. Discussion 

This scoping review of studies related to the governance of vaccine hesitancy that were published in China between 2007 and September 2019 is the first of its kind to the best of our knowledge. The findings indicate that most studies on vaccine hesitancy have defined it as a problem related to vaccine incidents and vaccine safety. A smaller number of studies have defined it as a problem related to professional conduct, and a very small number have defined it as a problem related to parental beliefs or cognitions. Accordingly, most studies have assigned the responsibility for vaccine hesitancy to governance system factors, such as an inadequate supervision model and reduced participation and transparency. As solutions, they have proposed reformation of the supervision model, a strict top-down accountability system, and participatory turn in crisis response. A handful of studies have ascribed vaccination hesitancy to less responsive and less experienced doctors and relatively outdated technical equipment at the grassroots level. Professional training, resource investment, and regulation of doctors have been called for as solutions. Studies that focused on parental doubts and beliefs pointed to the influence of the media and inadequate public education. These studies proposed public outreach and communication as solutions. A few studies evaluated the new policies formulated to tackle this problem and pointed to diverse factors that hampered the effective implementation of these policies.

Vaccination hesitancy is a global phenomenon. However, in a systematic review [5], it was concluded that the global determinants of vaccination hesitancy are country- and context-specific. In line with this, researchers [6] have demonstrated that immunization managers in different global regions have identified diverse locally relevant factors: religion, culture, socioeconomic situation, influential leaders and anti-vaccination lobbies, geographic barriers, personal risk perception and knowledge, introduction of a new vaccination, hesitancy among healthcare workers and among migrants, cost of vaccines, and role of healthcare professionals. Both studies have argued that understanding the contextual factors is important for developing adequate strategies to reduce vaccine hesitancy [5,6]. This review underlines this insight by demonstrating the context-specific characteristics of vaccination hesitancy in China. Although many of the global determinants apply to the Chinese context, most studies have emphasized vaccine-related factors for hesitancy: Hesitance was mostly found to be associated with the safety of vaccines in the incidents that resulted in the death of children. Unsurprisingly, scholars have stressed the importance of reforming the model for supervising vaccine safety in China [30,31,32,44,45,46,47,48,49], participation of stakeholders in the governance of safety [29,30,31,43,44,45,46,47,48,55], more effective communication after several vaccine scandals have induced hesitancy [10,25,58], and raising the standards of medical doctors [65,87,88].

As such, the results of this review differ from the findings of another systematic review of vaccine hesitancy studies published between 2007 and 2012 [5]. The previous review of vaccine hesitancy in the West Pacific Region, the region in which China is located, was related mainly to specific socioeconomic backgrounds and personal beliefs, as well as attitudes and knowledge pertaining to vaccination, not to vaccine safety [5]. In our review, it appears that many academics and professionals in China consider vaccine hesitancy in relation to concerns about vaccine safety and its governance and supervision model. This difference in outcomes can possibly be explained by the fact that not many Chinese studies were included in [98], which, in turn, may be ascribed to the selected time period: Vaccine hesitancy in China was not studied extensively before 2012. It became a major problem only after a series of vaccine safety scandals and incidents in the last decade. This specific Chinese context helps us understand why a large number of studies by Chinese scholars have addressed vaccine hesitancy in relation to vaccine safety, the governance of vaccine safety, and the quality of healthcare professionals. Along similar lines, to reduce vaccine hesitancy and to increase public trust, most studies have pointed to improvements to the governance and supervision systems of vaccines in China. Few studies have dealt with parental beliefs and cognitions, but even most of these studies consider the beliefs of parents in the context of severe vaccination incidents.

**Figure 3:**
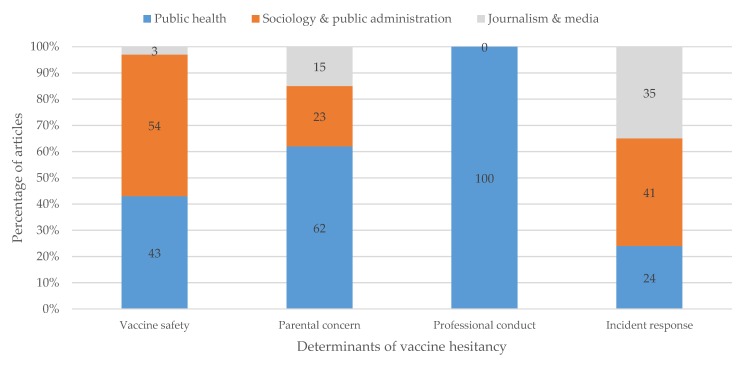
Relationship between categories of vaccine hesitancy and disciplinary backgrounds of scholars. Categories were assigned based upon problem articulation, solution proposals, and the allocation of responsibility (see Section 3.2 and Figure 2). Disciplinary background is drawn from author affiliations and personal information, also listed in Table A1.

Interestingly, our review revealed that the disciplinary backgrounds of scholars influence how they frame vaccination hesitancy. It appears that 54% of sociology and public policy experts, as well as 43% of public health experts tie vaccination hesitancy to vaccine safety and the governance of vaccine safety. A total of 41% of sociology and public policy experts, as well as 36% of journalists, mentioned inappropriate crisis management and a lack of participatory governance turns as the determinants of vaccine hesitancy. All the public health experts position vaccine hesitancy as a problem related to unprofessional conduct by healthcare professionals and parental beliefs (see Figure 3).

### Strengths and Limitations of Our Research

The adoption of a scoping review methodology enabled us to present an overview of Chinese studies on vaccine hesitancy and vaccine hesitancy governance. Moreover, we identified a relationship between the expertise of scholars and the way they defined vaccine hesitancy as a problem, as well as the routes to reduce vaccine hesitancy. Our study findings should be considered in the light of certain limitations. First, we reviewed publications from 2007 to September 2019, and we may have overlooked important studies published before 2007. However, there are indications that vaccine hesitancy was not a public problem in China before 2007. By then, China had not experienced severe vaccine incidents, and most people readily accepted immunization after witnessing the impact of infectious diseases, such as SARS in 2003 and avian influenza in 2004 [99]. The social memory of such epidemics intensified the public’s sense of human vulnerability and generated in the public a fear of future infectious disease, which spurred the public to vaccinate their children. Second, the fact that we identified 20 articles only after bibliography screening displays that there is a high terminological diversity at play in issues of vaccine hesitancy in the Chinese databases (e.g., “vaccine circulation”). Over time, as this resource is used more and links between Western and Chinese debates are intensified, we expect higher terminological standardisation. Careful bibliographic screening help reveal this, but it is possible that a small number of studies nonetheless were not caught. Given the scoping nature of this review, associated methodological risks are limited, but for systematic reviews using these databases, this is a concern. Finally, we solely focused on scholarly arguments from four Chinese databases and excluded public opinions and grey literatures. To obtain additional insights into the specific Chinese context of vaccination hesitancy, studies in the future should glean data about the narratives of the public’s concerns related to vaccine governance.

## 5. Conclusions

Chinese scholars primarily defined vaccine hesitancy as a vaccine safety problem and only secondarily as an issue of professional medical conduct or parental beliefs. Consequently, they primarily locate accountability on the level of governance and only secondarily at the level of public communication and media. The analysis suggests that strategies to mitigate public distrust in vaccination programs should not only be limited to education of, communication with, and information dissemination to parents but should also emphasize vaccine safety control, social participation, and transparency in vaccination governance, as well as raising the standards of medical professionals engaged in vaccination. Globally, most strategies to deal with vaccination hesitancy focus on the users of vaccination. However, in the Chinese context, scholars stress the importance of improving the vaccination program itself. 

## Figures and Tables

**Table 1:** Selection criteria for study inclusion and exclusion.

Inclusion	Exclusion
1. Scholarly peer-reviewed articles, conference papers, government reports, media reports. 2. Papers focused on governance of vaccination and public trust.	1. Publications that only mentioned vaccine safety in the conclusions. 2. Papers focused on preclinical medicine and veterinary medicine research.

## Appendix A

**Table A1:** Overview of selected research.

Article (*n* = 77)	Expertise	Perspective
Chen T, 2017. [1]	Public health.	Professional conduct
Liu, X., Hu W. & Zhang S, 2018. [10]	Sociology and Public administration (Public Administration).	Vaccine safety
Zhang Y, 2017. [12]	Public health (CDC).	Parental concern
Tong X, 2019. [14]	Sociology and Public administration (Public Administration).	Vaccine safety
Zhou Q, Liu W, Chen L, 2018. [15]	Public health.	Vaccine safety
Peng Z, Wang D, Yang J, 2018. [16]	Public health (Epidemiology).	Parental concern
Wang & He, 2016. [25]	Sociology and Public administration (Sociology).	Vaccine safety
Hu Y, 2014. [26]	Sociology and Public administration (Public Administration).	Vaccine safety
Liu X, Lin R, Yang C, Yu S, Zhang B, 2017. [29]	Public health.	Vaccine safety
CAMG, 2018. [30]	Sociology and Public administration (Sociology).	Vaccine safety
Li W, Chen W, Zhang J, 2016. [31]	Journalism and Media.	Vaccine safety
Sun Y, Xu L, Li S, 2015. [32]	Public health.	Incident response
Chen W, Gao Z, Li Y, 2016. [33]	Public health (epidemiology).	Vaccine safety
Di W, 2015. [34]	Sociology and Public administration (Sociology).	Vaccine safety
Shi L, 2017. [35]	Public health (CDC).	Vaccine safety
Sun W, 2014. [36]	Public health (Epidemiology).	Parental concern
Yu, W, et al., 2014. [37]	Public health (CDC).	Vaccine safety
Yuan & Li, 2017. [38]	Public health.	Vaccine safety
Zhang, K, 2017. [39]	Sociology and Public administration (Public Administration).	Parental concern
Ma J, Zhou L, Zhou L, 2015. [40]	Public health (CDC).	Vaccine safety
Meng &Xu, 2012. [41]	Sociology and Public administration (Public Administration).	Vaccine safety
Lu Y, 2018. [42]	Sociology and Public administration (Public Administration).	Vaccine safety
Wang & Yang, 2016. [43]	Public health (CDC).	Vaccine safety
Qian & Wang, 2012. [44]	Sociology and Public administration (Public Administration).	Vaccine safety
Zhang Y, 2014. [45]	Sociology and Public administration (Sociology).	Vaccine safety
Zhou& Li, 2014. [46]	Sociology and Public administration (Public Administration).	Vaccine safety
Jiang Y, Yu W, Zhang X, 2014. [47]	Public health (CDC).	Vaccine safety
Li H, 2019. [48]	Journalism and Media.	Incident response
Li & Chen, 2011. [49]	Sociology and Public administration (Public Administration).	Vaccine safety
Zhang H, 2018. [50]	Sociology and Public administration (Public Administration).	Vaccine safety
Han J, Zhou W, 2016. [51]	Sociology and Public administration (Sociology).	Incident response
Lai S, 2013. [52]	Sociology and Public administration (Public Administration).	Incident response
Qi &Cheng, 2015. [53]	Sociology and Public administration (Sociology).	Incident response
Sui X, 2014. [54]	Journalism and Media.	Incident response
Xiao X, 2017. [55]	Sociology and Public administration (Sociology).	Incident response
Song J, 2018. [56]	Journalism and Media.	Incident response
Song W. 2018. [57]	Journalism and Media.	Incident response
Huang, 2010. [58]	Sociology and Public administration (Public Administration).	Vaccine safety
Zhao D, Li X, Lu L, 2018. [59]	Public health.	Professional conduct
Xiang F, 2012. [60]	Public health.	Incident response
Cheng M, Su Z, Lian Q, 2014. [61]	Public health.	Professional conduct
Liu F, 2014. [62]	Public health.	Professional conduct
Wang Y, 2012. [63]	Public health.	Professional conduct
Guo W, Wang J, Yu X, 2018. [64]	Public health.	Professional conduct
Qiao X, Wei Ji, Lu D, 2018. [65]	Public health (Epidemiology).	Professional conduct
Zhao X, Zhou L, Yang X, 2016. [66]	Public health.	Professional conduct
An L, Chen W Sun M, 2018. [68]	Public health.	Parental concern
Tang Z, 2018. [69]	Public health (Epidemiology).	Parental concern
Dai & Zhu, 2018. [70]	Journalism and Media.	Parental concern
Yang H,2017. [71]	Sociology and Public administration (Sociology).	Vaccine safety
Cao L, Wang H, and Zheng, 2012. [72]	Public health (CDC).	Parental concern
Ma G, 2016. [73]	Sociology and Public administration (Sociology).	Parental concern
Yu F, 2016. [74]	Public health (Epidemiology).	Parental concern
Wang Y, Sun L, Li M, 2019. [75]	Public health.	Professional conduct
Zhuang X, Wang R, 2016. [76]	Public health (Epidemiology).	Parental concern
Huang S, 2015. [77]	Journalism and Media.	Parental concern
Kunming CDC, 2018. [78]	Public health (CDC).	Vaccine safety
Xue & Li, 2018. [79]	Sociology and Public administration (Public Administration).	Vaccine safety
Yu W, Ji S, Liu J, Cong B, Zhou Y, Zhang X, Cui F, Wang H, 2016. [80]	Public health (CDC).	Vaccine safety
Yi & Liao, 2013. [81]	Sociology and Public administration (Public Administration).	Vaccine safety
Zhang & Chen, 2010. [82]	Public health (CDC).	Incident response
Yang & Ding, 2014. [83]	Public health.	Professional conduct
Wu Z, 2013. [84]	Public health (CDC).	Vaccine safety
Wang B, 2018. [85]	Journalism and Media.	Incident response
Feng B, 2014. [86]	Public health.	Professional conduct
Wen W, 2011. [87]	Public health (CDC).	Vaccine safety
Zhong X, Lu Z, Chen X, 2017. [88]	Public health.	Professional conduct
Song J, 2015. [89]	Sociology and Public administration (Public Administration).	Incident response
Jia X, 2016. [90]	Sociology and Public administration (Sociology).	Incident response
Lai H, 2018. [91]	Sociology and Public administration (Sociology).	Parental concern
Sun L, Cong Y, Wang Y, 2017. [92]	Public health (CDC).	Incident response
Yue D, Chang J, Hou Z, Wu Q, Meng Y, 2014. [93]	Public health.	Vaccine safety
Ye and Zhang, 2019. [94]	Sociology and Public administration (Public Administration).	Vaccine safety
Sun L, Guo J, Li J, 2018. [95]	Public Health (CDC).	Vaccine safety
Zhao Z, 2019. [96]	Sociology and Public administration (Public Administration).	Incident response
Li T, 2011. [97]	Journalism and Media.	Incident response
Zhang Z, 2014. [98]	Sociology and Public administration (Public Administration).	Vaccine safety
